# *Babesia microti* (Babesiidae, Piroplasmida) infection in a Chinese traveler returning from the United States of America

**DOI:** 10.1186/s40249-025-01311-x

**Published:** 2025-06-10

**Authors:** Xin-An Huang, Rong Xiang, Ru-He Liao, Yu-Bo Luan, Yi-Lin Zhao, Ji-Hu Yang, Chun-Feng Luo, Lin Huang, Luo-Yuan Xia, Dai-Yun Zhu, Yi Sun, Lei Wang, Jia-Fu Jiang

**Affiliations:** 1 Artemisinin Research Center, Guangzhou University of Chinese Medicine, Guangzhou, 510000 People’s Republic of China; 2 State Key Laboratory of Pathogen and Biosecurity, Beijing Institute of Microbiology and Epidemiology, Academy of Military Medical Sciences, Beijing, 100071 People’s Republic of China; 3 Internal Medicine, Beijing United Family Hospital, Beijing, 100015 People’s Republic of China; 4 Beijing Institute of Tropical Medicine, Beijing Friendship Hospital, Capital Medical University, Beijing, 100050 People’s Republic of China; 5 Beijing Key Laboratory for Research On Prevention and Treatment of Tropical Diseases, Beijing, 100050 People’s Republic of China

**Keywords:** Human babesiosis, *Babesia microti*, Hemophagocytosis, Travel-related diseases

## Abstract

**Background:**

Human babesiosis, caused by intraerythrocytic protozoa of the genus *Babesia* (Piroplasmida, Babesiidae), is a globally emerging zoonosis transmitted primarily through *Ixodes* spp. ticks. *Babesia microti*, which is endemic particularly in the northeastern and midwestern United States, accounts for the majority of globally reported human cases. Recent studies highlight its spread to non-traditional regions and cross-border transmission, driven by climate change, blood transfusions and increased human mobility. Despite increasing reports of autochthonous *B. microti* infections in certain areas of China, imported cases remain critically underrecognized due to overlapping clinical manifestations with malaria and limited diagnostic awareness.

**Case presentation:**

We report a diagnostically challenging case of acute *B. microti* infection in a 52-year-old Chinese woman, presenting with a sudden recurrent fever (39.0–41.0 °C), hemolytic anemia (hemoglobin 104 g/L), thrombocytopenia (platelet 78 × 10^9^ /L) and splenic hypodense lesions on July 11, 2023, seven days after returning from a 14-day visit to rural Wisconsin, United States. Peripheral blood smears demonstrated characteristic intraerythrocytic ring forms (parasitemia: 7800 organisms/μl) and pathognomonic "Maltese cross" tetrads. Polymerase chain reaction (PCR) targeting the 18S rRNA gene confirmed *B. microti* infection (GenBank No. PP087232), showing 99.8% identity with the US-type strain Gray (AY693840) and the sequence obtained from a US travel-acquired case in Singapore (MK609547). The patient received intravenous clindamycin (600 mg twice daily), oral dihydroartemisinin (80 mg twice daily), packed red blood cell transfusions, and supportive care, ultimately achieving full recovery after 17 days.

**Conclusions:**

This study documented the first imported cases of human babesiosis in China, emphasizing the need for heightened clinical and public health vigilance. Screening travelers from endemic areas presenting with fever or hemolytic anemia for Babesia, bolstering molecular diagnosis, improving transfusion safety, and intensifying regional surveillance are crucial in reducing underdiagnosis and preventing transmission. These measures are essential for controlling babesiosis in China.

**Graphical Abstract:**

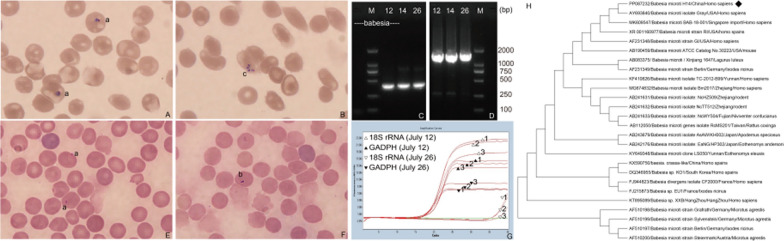

**Supplementary Information:**

The online version contains supplementary material available at 10.1186/s40249-025-01311-x.

## Background

Human babesiosis is a zoonotic disease primarily transmitted through tick bites and blood transfusions. The infection targets erythrocytes and exhibits a broad clinical spectrum, ranging from asymptomatic infection to life-threatening complications, with elderly patients and immunocompromised individuals at elevated risk for severe outcomes [1, 2]. Currently, only four *Babesia* species (*B. microti*, *B. duncani, B. divergens* and *B. venatorum*) and three similar sibling species (*B. divergens*-like, *B. crassa*-like and *B. microti-*like organisms) have been confirmed to cause human infections [2, 3]. Since the first reported human case of babesiosis in 1957, *Babesia* infections have been documented globally, with the highest prevalence in the United States of America (USA) [4], where the majority of cases are attributed to *B. microti* [5]. Recent studies highlight its spread to non-endemic regions driven by climate change and blood transfusion [6], and even cross-border transmission to Singapore due to increased patient mobility [7]. As of now, China has reported over 300 cases of symptomatic babesiosis, predominantly caused by *B. microti* in southern regions and *B. venatorum* in the northeast [8]. In 2024, a case was reported of a USA permanent resident who was initially diagnosed with pneumonia, transferred to a hospital in China for treatment on September 1, 2023, and later diagnosed with *B. microti* [9]. In this context, we present a case of Chinese woman infected with *B. microti*, confirmed on July 14, 2023, seven days after returning from a 14-day visit to rural Wisconsin, USA.

## Case presentation

### Patient history and presentation

A 52-year-old female patient from Beijing experienced a sudden-onset of high fever (39.0–41.0 °C) on July 11, 2023, just one week after returning from a two-week trip to Wisconsin in the USA (Additional file 1: Fig. S1). Upon admission, her blood tests showed thrombocytopenia (78 × 10^9^/L; normal range 125–350 × 10^9^/L), decreased erythrocyte count (3.3 × 10^12^/L; normal range 3.8–5.1 × 10^12^/L), and reduced hemoglobin (104 g/L; normal range 115–150 g/L), consistent with hemolytic anemia. Biochemical analysis revealed elevated levels of glutamic-pyruvic transaminase (107 U/L; normal range 7–40 U/L), glutamic oxalacetic transaminase (129 U/L; normal range 13–35 U/L), glutamyl transferase (83 U/L; normal range 7–45 U/L) and heightened C-reactive protein level (186 mg/L; normal range 0–10 mg/L) (Additional file 1: Fig. S2; Table S1). Abdominal CT scan also confirmed splenomegaly with multiple irregular hypodense lesions (Additional file 1: Fig. S3).

### Diagnostic confirmation

**Fig. 1:**
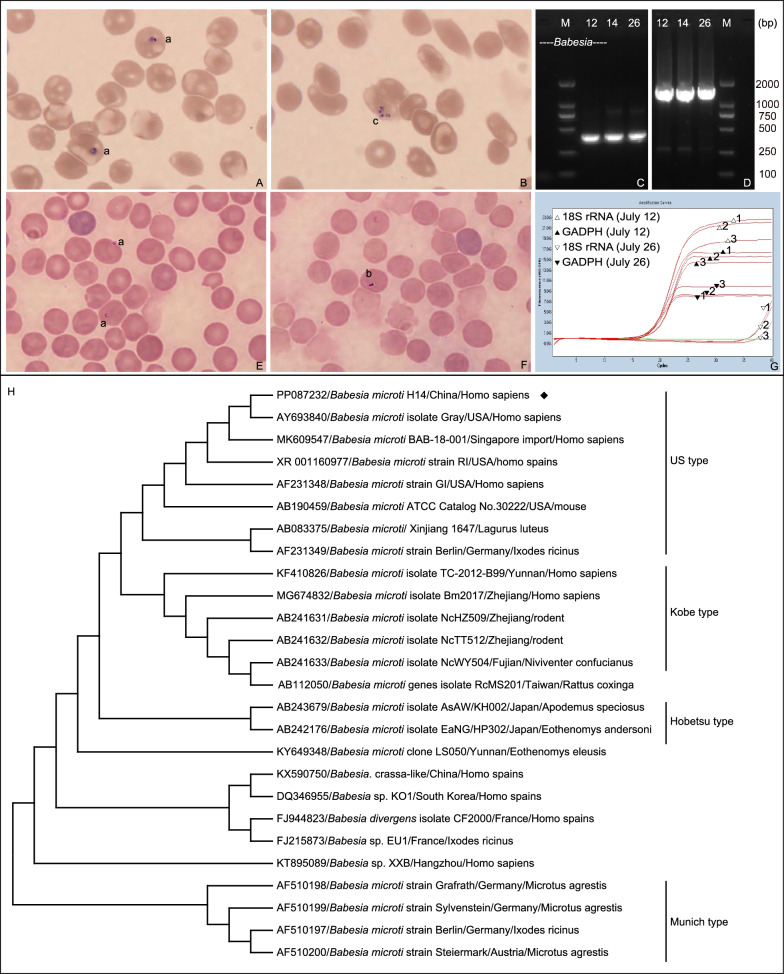
Peripheral blood smear photomicrographs depicting erythrocytes infected with *Babesia* spp. from both the patient and inoculated mice, complemented by molecular identification of the infecting parasite strain in the patient. **A**, **B** Initial presentation revealed typical ring-form trophozoites (a) and tetrameric (c) structures in the blood smear of the patient; **C** electrophoresis results displaying partial 18S rRNA target gene of *Babesia* spp. from human whole blood (Lanes 1–3). Lane 1, Lanes 2 and 3 show the amplification results on 12, 14 and 26 July, respectively. M means marker; **D** nested PCR product electrophoresis of covering the entire 18S rRNA gene from *B. microti*. Lane 1, Lanes 2 and 3 show the amplification results on 12, 14 and 26 July, respectively; **E**, **F** Giemsa-stained blood smears observed ring-form trophozoites (a) at day 3 post-inoculation and paired pyriform merozoites (b) at day 9; **G** Real-time fluorescence quantitative PCR depicting changes in *B. microti* load. Each sample was replicated three times, denoted by 1, 2, 3; **H** Comparative analysis of the 1667 base-pair nucleotide sequence alignment of the *Babesia* 18S rRNA target gene. The black icon indicates the sequence of this study. Phylogenetic analysis was performed using the neighbor-joining method and trees were tested by bootstrapping (1000 replicates)

Giemsa-stained blood smears revealed typical erythrocyte ring trophozoites and tetrameric structures (Fig. 1A, B). Parasitemia was quantified at 7800 organisms/μl on July 14, 2023 with diagnostic procedures following WHO 2015 standardized protocols for blood film preparation and malaria parasite counting [10]. Subsequently, DNA was extracted from the blood and subjected to screening for *Babesia* and *Plasmodium* using specific primers via nested PCR [11], as well as for other potential pathogens, including Lyme disease, scrub typhus, sepsis, and *Mycobacterium tuberculosis* (Additional file 1: Table S2). The results revealed the presence of only *Babesia* spp. (Fig. 1C). Consequently, the patient was confirmed as having human babesiosis, aligning with the criteria recommended by the US Centers for Disease Control and Prevention [5].

### Treatment and response

Before receiving definitive diagnosis, the patient was treated empirically with doxycycline (100 mg twice daily) and amoxicillin (500 mg every four hours) for 48 h. According to the 2020 Infectious Diseases Society of America guidelines, the recommended treatment for human babesiosis involves the use of atovaquone and azithromycin [12]. However, atovaquone was not immediately available in Beijing due to supply constraints. Based on the documented antiparasitic efficacy of artemisinin and its successful use at our hospital, combination therapy with intravenous clindamycin (600 mg twice daily) and oral dihydroartemisinin (80 mg twice daily) for 14 days was initiated [13]. Supportive care included infusion of human albumin, packed red blood cell transfusion, and antipyretic therapy (Additional file 1: Table S3). After 72 h of combination therapy, the patient showed a gradual recovery (Additional file 1: Table S4, Fig. S2). At the later stages of the treatment (July 26), the blood smear result turned negative, while the nested PCR result remained positive (Fig. 1C). The patient was discharged on July 28, 2023.

### In vivo isolation and real-time quantitative PCR verification

Subsequently, blood samples from the patient (200 μl per mouse) were intraperitoneally injected into five 3-week-old male mice (SCID, Vital River, Beijing). Tail blood was collected every 3 days for Giemsa staining, microscopic analysis, and PCR. Intraerythrocytic *Babesia* parasites were detected in blood smears at days 3 and 9 post-inoculation (Fig. 1E, F). Additionally, quantitative real-time PCR using human housekeeping genes, specifically Glyceraldehyde-3-phosphate dehydrogenase as the endogenous control revealed a 3.29 × 10^6^-fold reduction in *B. microti* DNA load between July 12 and July 26 (Fig. 1G, Additional file 1: Table S5). Post-discharge monitoring until August 11, 2023 confirmed complete recovery, demonstrated by consecutive negative blood smears and PCR assays.

### Genetic and phylogenetic insights

An amplification of the full-length 18S rRNA gene was conducted and successfully obtained (Fig. 1D) as per the previous report [14]. The nearly complete 18S rRNA sequence (1667 bp, GenBank accession No. PP087232) revealed a remarkable 99.88% similarity with the *B. microti* isolated from a patient in the USA (GenBank accession no. AY693840) and a US travel-acquired patient from Singapore (MK609547). The nucleotide sequence exhibited 11 base pair variations (99.0% identity) when compared to the common Kobe-type Babesia microti strain (GenBank accession no. KF410826) isolated from Chinese patients (Fig. 1H, Additional file 1: Fig. S4).

## Discussion and conclusions

Although human babesiosis is uncommon in most regions of China, increasing evidence points to significant underdiagnosis of human cases [8]. Notably, the case presented here is the confirmed instance of first imported case of babesiosis from abroad.

Babesiosis presents with clinical symptoms and biological abnormalities that are similar to those observed in *Plasmodium* infection. After ruling out malaria, clinicians should remain highly vigilant for other intraerythrocytic protozoan infections. In Europe, *B. divergens* and *B. venatorum* predominate, whereas in North America, *B. microti* is responsible for most human babesiosis cases [15]. Microscopic examination of Giemsa-stained blood smears remains the traditional method for diagnosing babesiosis, revealing the presence of intraerythrocytic parasites. Diagnostic features include ring-form trophozoites, tetrads, or paired pyriform parasites. In this case, common PCR targeting 18S rRNA confirmed the diagnosis of babesiosis and quantitative real-time PCR tested very low parasite loads. PCR-based confirmation is recommended due to its superior sensitivity and specificity compared to blood smears [16].

Wisconsin, where approximately 46% of the land is covered in forests and around 9% of these forests are part of the Chequamegon-Nicolet National Forest, ranks among the 14 states in the USA with a high prevalence of tick-borne infections [17]. From 2011 to 2020, a total of 635 babesiosis cases were reported in this state [5]. In contrast, only two babesiosis cases have been reported in Beijing, China to date, with no conclusive reports of *B. microti*-infected patients [8]. The present patient traveled to and visited the Chequamegon-Nicolet National Forest. Genetic analysis of the obtained sequences confirmed her infection with the US type of *B. microti*, which is distinct from the Kobe strain identified in indigenous Chinese *B. microti* cases. It's worth noting that patients often remain unaware of tick bites; a review of 38 patients in the Upper Midwest, USA, revealed that 55% did not recall being bitten by a tick [18]. This present case strongly suggests that human babesiosis can be acquired through travel, highlighting international travel as a significant pathway for importing babesiosis from endemic regions. Coincidentally, shortly after, a 67-year-old Chinese-American man, long settled in the USA, developed persistent fever and was diagnosed with pneumonia in the USA on August 29, 2023. He immediately went to Zhuhai city, China for further treatment, where he was unexpectedly confirmed to be infection with *B. microti* [9]. However, due to the absence of shared genetic sequencing data, we are unable to perform a genotype–phenotype correlation analysis with this patient. Anyway, both cases underscore international travel as a key risk factor for importing babesiosis.

In the case presented in this study, the treatment approach involved the intravenous administration of clindamycin, oral intake of dihydroartemisinin, packed red blood cell transfusions, and supportive care. Currently, in China, there is no established standard treatment protocol for human babesiosis. Across various hospitals, clindamycin, azithromycin, atovaquone, and doxycycline are frequently used to treat human babesiosis [9, 19–21]. Generally, the typical treatment regimen combines pharmacotherapy with supportive care. Therefore, it is urgently necessary to further explore more effective and standardized treatment strategies to address similar cases that may arise in the future.

In conclusion, this study documented the first imported cases of human babesiosis in China, emphasizing the urgent need for increased clinical and public health vigilance. It is imperative to prioritize *Babesia* screening for travelers from endemic regions who exhibit symptoms such as fever or hemolytic anemia. Strengthening molecular diagnostic techniques, optimizing transfusion safety protocols (e.g., donor screening), and enhancing regional surveillance are pivotal strategies for reducing underdiagnosis rates and preventing secondary transmission within at-risk areas. These measures are essential for effectively managing and controlling the spread of babesiosis in China.

## Supplementary Information

Additional file 1: Fig. S1. Timeline for the patient infected with *Babesia microti *(Piroplasmida, Babesiidae) and the clinical course and treatment. Fig. S2. Laboratory values observed during hospitalization. The x-axis denotes the timeline in dates, and the y-axis displays quantitative measurements of laboratory parameters. The area bounded by red dashed lines represents the reference range. The continuous trend line plots the variations in the patient's laboratory parameters over time. Blue lines indicate normal values, and red lines indicate abnormal values. WBC: white blood cell; RBC: red blood cell; HGB: hemoglobin; PLT: platelet; CRP: C-reactive protein; AST: glutamic oxalacetic transaminase; ALT: glutamic-pyruvic transaminase; GGT: glutamyl transferase. Fig. S3. Abdominal CT scan demonstrating massive splenomegaly. The red and blue lines indicate the length and width of the spleen, respectively. The red line (12.5 cm) exceeds the upper limit of normal splenic length (10 cm); the blue line (7.4 cm) surpasses the normal transverse diameter (6 cm). Fig. S4. Comparison analysis of the 1667 base-pair nucleotide sequence alignment of Babesia 18S rRNA gene. The first sequence was from the patient in this study. The other five sequences were obtained from GenBank. Table S1. Laboratory values observed during the patient's hospitalization (reference values in parentheses). Table S2. The panel assays screen for six common viruses, bacteria and protozoa. Table S3. Supportive treatment regimen. Table S4. Symptoms and signs reported during the hospital stay. Table S5. Changes in Babesia microti load under real-time fluorescence quantitative PCR.

## Data Availability

The study datasets are available from the corresponding author and can be shared upon reasonable request.

## Abbreviations

USA: United States of America
PCR: Polymerase chain reaction
rRNA: Ribosomal ribonucleic acid
RBC: Red blood cell
