# Supplementary material for: Babesia microti (Babesiidae, Piroplasmida) infection in a Chinese traveler returning from the United States of America
Source: Infect Dis Poverty. 2025 Jun 10;14:45. doi: 10.1186/s40249-025-01311-x (PMC12150541; doi:10.1186/s40249-025-01311-x)
Supplement: Supplementary file 1 — Additional file 1: Fig. S1. Timeline for the patient infected with Babesia microti (Piroplasmida, Babesiidae) and the clinical course and treatment. Fig. S2. Laboratory values observed during hospitalization. The x-axis denotes the timeline in dates, and the y-axis displays quantitative measurements of laboratory parameters. The area bounded by red dashed lines represents the reference range. The continuous trend line plots the variations in the patient's laboratory parameters over time. Blue lines indicate normal values, and red lines indicate abnormal values. WBC: white blood cell; RBC: red blood cell; HGB: hemoglobin; PLT: platelet; CRP: C-reactive protein; AST: glutamic oxalacetic transaminase; ALT: glutamic-pyruvic transaminase; GGT: glutamyl transferase. Fig. S3. Abdominal CT scan demonstrating massive splenomegaly. The red and blue lines indicate the length and width of the spleen, respectively. The red line (12.5 cm) exceeds the upper limit of normal splenic length (10 cm); the blue line (7.4 cm) surpasses the normal transverse diameter (6 cm). Fig. S4. Comparison analysis of the 1667 base-pair nucleotide sequence alignment of Babesia 18S rRNA gene. The first sequence was from the patient in this study. The other five sequences were obtained from GenBank. Table S1. Laboratory values observed during the patient's hospitalization (reference values in parentheses). Table S2. The panel assays screen for six common viruses, bacteria and protozoa. Table S3. Supportive treatment regimen. Table S4. Symptoms and signs reported during the hospital stay. Table S5. Changes in Babesia microti load under real-time fluorescence quantitative PCR. [file 40249_2025_1311_MOESM1_ESM.pdf]

Additional file 1 for:

***Babesia microti* (Babesiidae, Piroplasmida) infection in a Chinese traveler returning from the United States of America**

Xin-An Huang[1*], Rong Xiang[2*], Ru-He Liao[1,2*], Yu-Bo Luan[3*] , Yi-Lin Zhao[2], Ji-Hu Yang[2], Chun-Feng Luo[2], Lin Huang[2], Luo-Yuan Xia[2], Dai-Yun Zhu[2], Yi Sun[2], Lei Wang[4,5†], Jia-Fu Jiang[1,2†]

[1] Artemisinin Research Center, Guangzhou University of Chinese Medicine, Guangzhou 510000, P.R. China

[2] State Key Laboratory of Pathogen and Biosecurity, Beijing Institute of Microbiology and Epidemiology, Academy of Military Medical Sciences, Beijing 100071, P.R. China

[3] Internal Medicine, Beijing United Family Hospital, Beijing, 100015, P. R. China

[4] Beijing Institute of Tropical Medicine, Beijing Friendship Hospital, Capital Medical University, Beijing 100050, P. R. China

[5] Beijing Key Laboratory for Research on Prevention and Treatment of Tropical Diseases, Beijing 100050, P. R. China

[*]Contributed equally.

[†] Contributed equally.

Correspondence: Jia-Fu Jiang (jiangjf2008@139.com); Lei Wang (wangleibfh@ccmu.edu.cn).

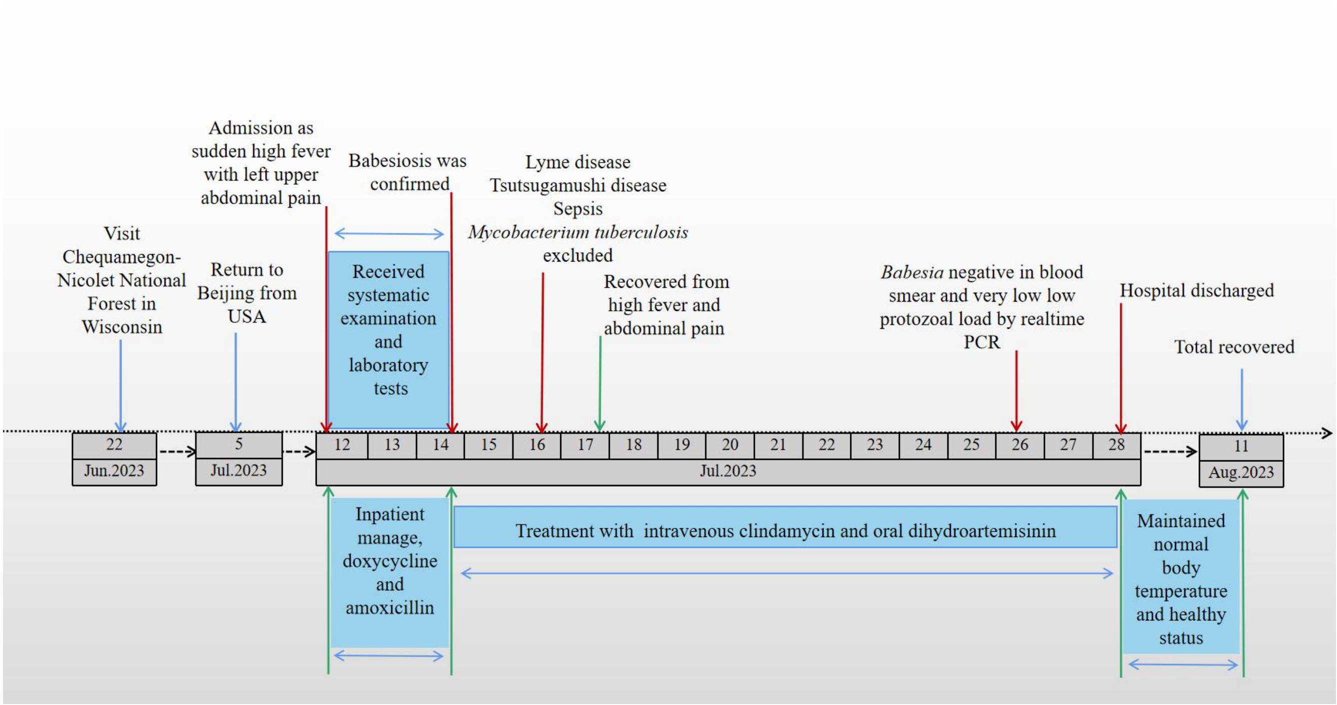

Figure S1: Timeline for the patient infected with *Babesia microti* (Piroplasmida: Babesiidae) and the clinical course and treatment.

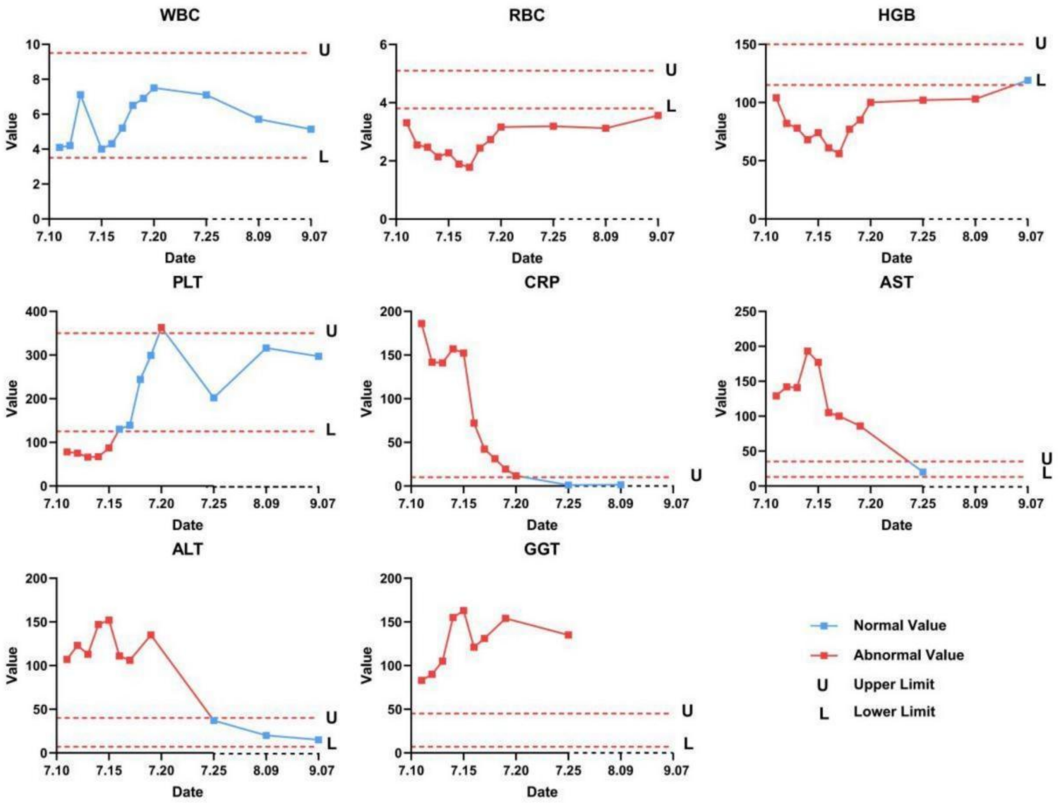

Figure S2: Laboratory values observed during hospitalization. The x-axis denotes the timeline in dates, and the y-axis displays quantitative measurements of laboratory parameters. The area bounded by red dashed lines represents the reference range. The continuous trend line plots the variations in the patient's laboratory parameters over time. Blue lines indicate normal values, and red lines indicate abnormal values. WBC: white blood cell; RBC: red blood cell; HGB: hemoglobin; PLT: platelet; CRP: C-reactive protein; AST: glutamic oxalacetic transaminase; ALT: glutamic-pyruvic transaminase; GGT: glutamyl transferase.

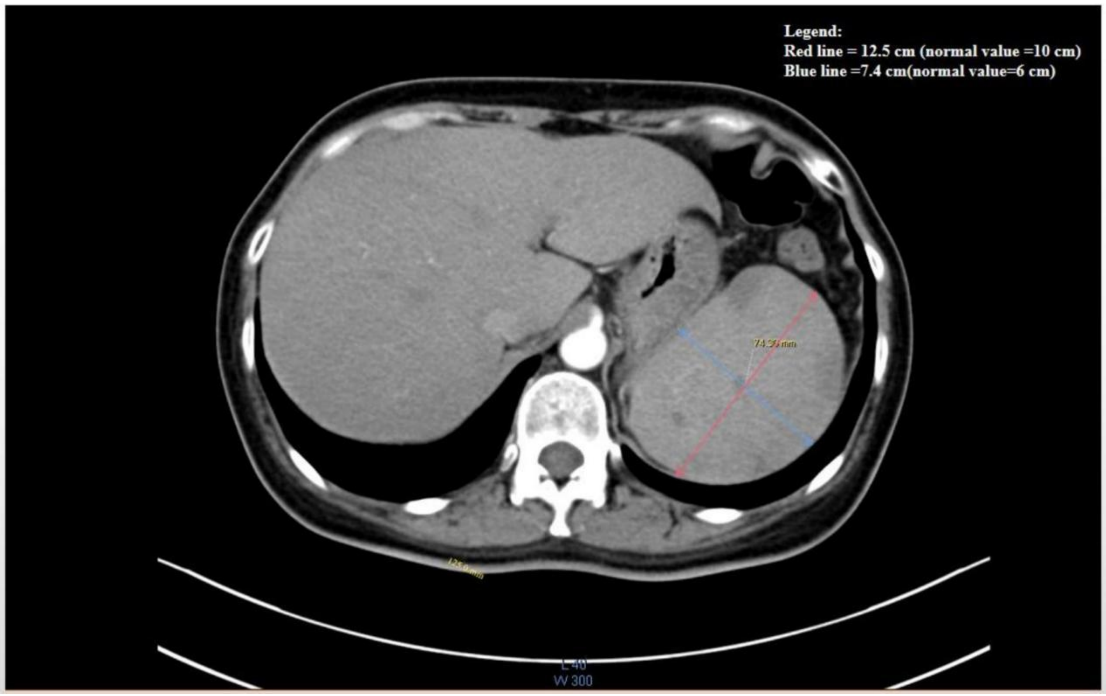

Figure S3: Abdominal CT scan demonstrating massive splenomegaly. The red and blue lines indicate the length and width of the spleen, respectively. The red line (12.5 cm) exceeds the upper limit of normal splenic length (10 cm); the blue line (7.4 cm) surpasses the normal transverse diameter (6 cm).

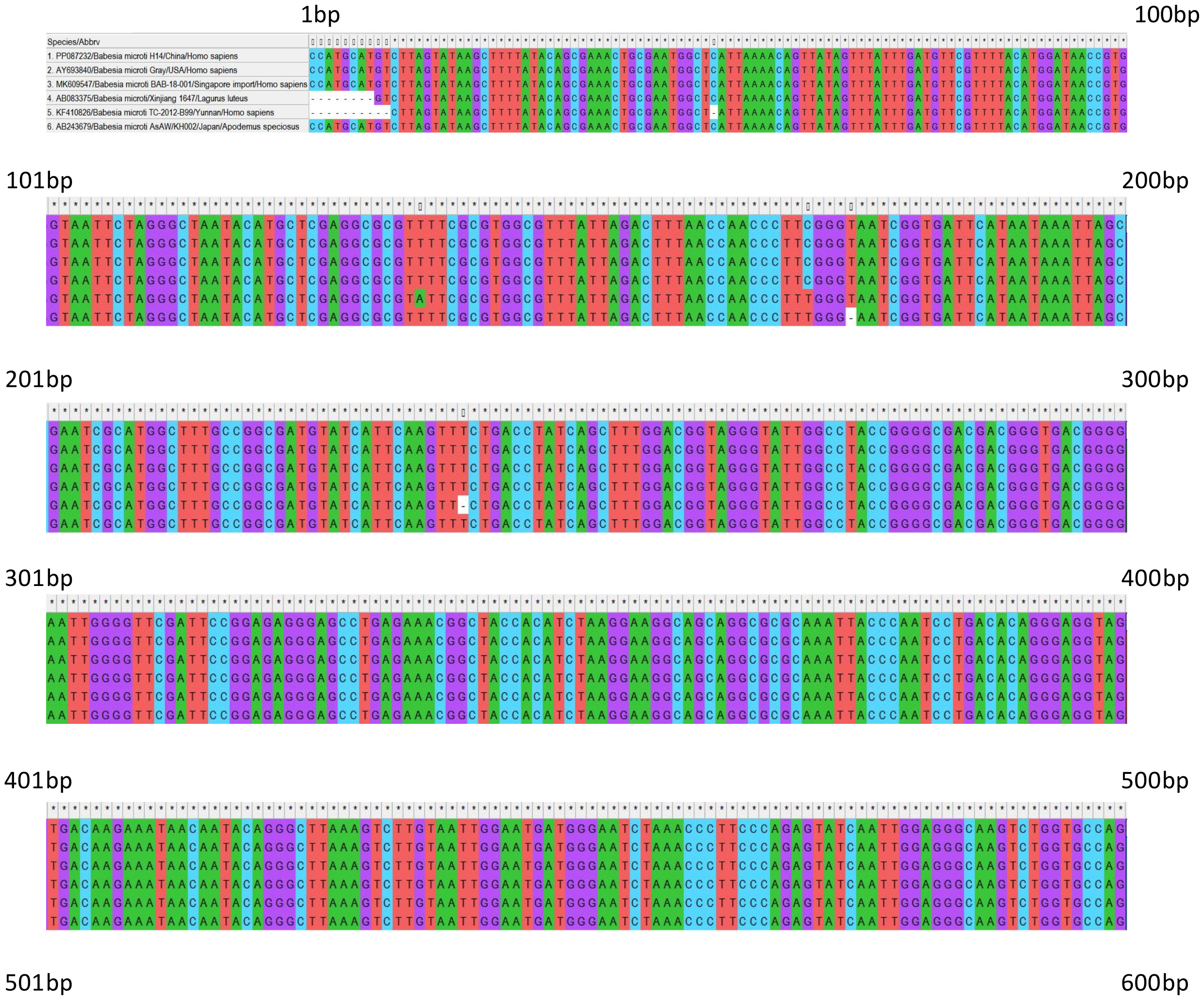

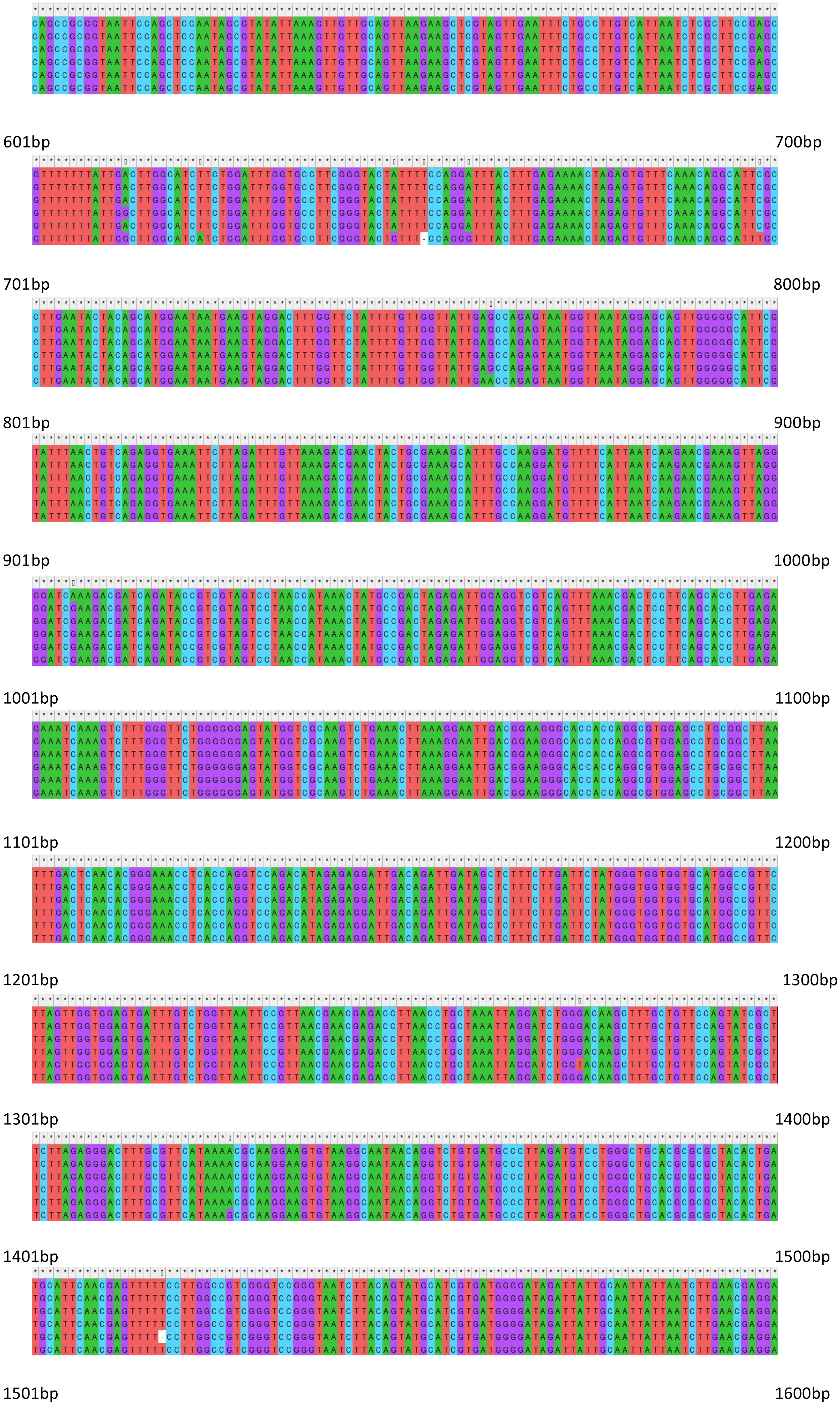
601bp
700bp
701bp
800bp
801bp
900bp
901bp
1000bp
1001bp
1100bp
1101bp
1200bp
1201bp
1300bp
1301bp
1400bp
1401bp
1500bp
1501bp
1600bp

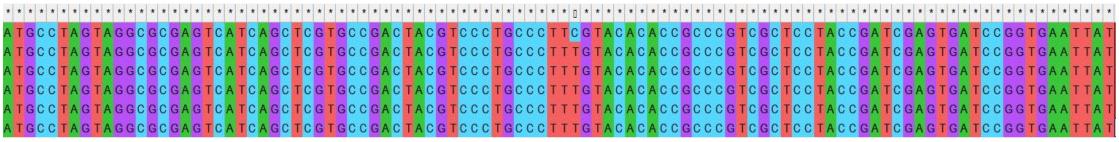

1601bp 1667bp

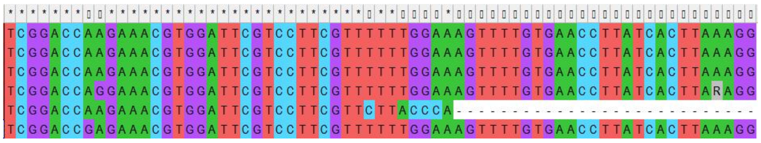

Figure S4: Comparison analysis of the 1667 base-pair nucleotide sequence alignment of *Babesia* 18S rRNA gene. The first sequence was from the patient in this study. The other five sequences were obtained from GenBank.

Table S1. Laboratory values observed during the patient's hospitalization (reference values in parentheses)

**Date of testing**	**2023–7–11**	**2023–7–12**	**2023–7–13**	**2023–7–14**	**2023–7–15**	**2023–7–16**	**2023–7–17**	**2023–7–18**	**2023–7–19**	**2023–7–20**	**2023–7–25**
White blood cell (3.5–9.5×10^9 /L)	4.1	4.2	3.5	3.2	4	4.3	5.2	6.5	6.9	7.5	7.1
Neutrophil (0.4–0.75%)	37.40%	46.40%	42.20%	49.90%	58.70%	56.40%	54%	59.50%	55.10%	52.30%	63.60%
Lymphocyte count (1.1–1.3×10^9 /L)	2	1.8	1.7	1.3	1.4	1.6	2	2.2	2.3	2.4	1.5
Hemoglobin (115–150 g/L)	104	81	78	68	74	61	56	77	85	100	102
Red blood cell (3.80–5.10×10^{12} /L)	3.31	2.55	2.47	2.14	2.28	1.89	1.78	2.44	2.73	3.16	3.19
Platelet (125–350×10^9 /L)	78	75	66	67	87	130	139	244	299	363	202
Infected red cells			0.2%-0.9%	1%		≤0.1%		0	0	0	
C reaction protein (0.0–10.0 mg/L)	186	141.7	141	157.1	152.3	72	42.3	31.2	19.4	11.7	1.1
Procalcitonin (<0.05 ng/ml)			0.546		0.356	0.348					0.159
Ferritin (15–200 ng/ml)			3318.7								
IL-6 (<5.5 pg/mL)			119.08		19.47	23.27					9.42
Sodium (137.0–147.0 mmol/L)	138	136	137.7	138.1	141.9	141.5	139.7	137.8		134.1	134.9
Potassium (3.5–5.5 mmol/L)	3.5	3.2	3.6	3.2	4.3	4	3.8	4.5		4.3	4.5
Chloride (99.0–110.0 mmol/L)	103	102	106.8	105	108.6	108.5	105.7	103.8		97.1	102.1
Urea nitrogen (2.6–7.5 mmol/L)	4.5	3.2	2.1	2.5	2.9	4.1	4.4	5.3		6.8	9.4
Creatinine (41–73 µmol/L)	58	56	50	50	49	57	56	58		69	94
Calcium (2.11–5.52 mmol/L)	2.15	2.04	1.84	1.95	2.04	1.99	2.04	2.15		2.35	2.26
Phosphorus (0.85–1.51 mmol/L)	0.92	0.84			1.07	0.99	1.1				0.89
Uric acid (178–406 µmol/L)	268	256					209				248
Bilirubin total (5.0–21.0 µmol/L)	27.3	29.6	23.8	32.7	32.2	22.4	14.5		13.4		12.9
Bilirubin direct (1.7–12.4 µmol/L)		17	11.9	17.4	18.5	15.6					
AIKP (50–135 U/L)	125	130	135	207	217	153	148		149		121
AST (13–35 U/L)	129	142	141	193	177	105	100		86		20
ALT (7–40 U/L)	107	123	113	147	152	111	106		135		37
GGT (7–45 U/L)	83	90	105	155	163	121	131		154		135
Albumin (40–55 g/L)	38	33	26	29	31	30	33		43		48
Glucose Random(3.8–7.8 mmol/L)	5.4	5.9	5.7	6.5	8.3	5.4	4.9	5.1		4.7	5.9
Prothrombin time (10.0–12.8 seconds)		12.5			12.2		11.2				
Activated partial thromboplastin time (22.0–35.0 seconds)		31.2			35.8		24.3				
INR (0.8–1.2 seconds)		1.11			1.08		0.98				
Fibrinogen (2.0–4.0 g/L)		4.76			4.32						
NT-BNP (<169 pg/mL)					2998	1866	1831				

AIKP: Alkaline phosphatase; AST: Aspartate aminotransferase; ALT: Alanine aminotransferase; GGT: Gamma-glutamyl transpeptidase; INR: International Normalized Ratio; NT-BNP: N terminal pro B type natriuretic peptide.

Table S2: The panel assays screen for six common viruses, bacteria and protozoa

Screening approach	Test kit	Methods
Lyme disease	The Lyme disease BB antibody protein blot assay (IgG detection kit for anti-Spirospira antibody, Scientific research reagent, DN 2131-3201 G LS)	The PCR-fluorescence probe method
Scrub typhus	The Scrub Typhus IgG/IgM antibody assay (IgG/IgM Rapid Test Kit for anti- *Orientia tsutsugamushi* structural protein, F1406222)	Immunochromatographic method
Sepsis	Blood culture (Aerobic bacteria, anaerobic bacteria and fungi culture, XJ7251)	Culture
Mycobacterium tuberculosis	Mycobacterium tuberculosis protein MPB64 (TB specific sequence kit for Mycobacterium tuberculosis complex, FZ2031102)	Qualitative real-time PCR
Plasmodium	DreamTaq Green PCR Master Mix(JK20230714)	Nested PCR
Babesia	DreamTaq Green PCR Master Mix(JK20230714)	Nested PCR

Table S3. Supportive treatment regimen

Pharmaceutical agents	Daily dosage	Duration	Delivery route
Human albumins	10 g	7 days	intravenous
Blood products (suspended red blood cells)	200 mL	3 days	intravenous
Antipyretics (lysine Acetylsalicylate/Diclofenac Sodium)	0.9 g/0.075g	5 days (temperature above 38.5 ℃)	intravenous/oral

Table S4. Symptoms and signs reported during the hospital stay

Dates	Temperature (°C)	Arterial pulse （time/ minute）	Blood pressure （mmHg）	Respire (time/ min ute)	Head ache	Fatigue	Abdomi nal pain	Dizziness
2024–7–11 (Day 1)	38	94	110/73	20	+	+	+	-
2024–7–12 (Day2)	39.5	112	108/72	17	+	+	+	+
2024–7–13 (Day 3)	39.4	98	99/61	20	+	+	+	+
2024–7–14 (Day 4)	38.7	103	102/65	20	+	+	+	-
2024–7–15 (Day 5)	37.2	84	107/70	18	-	+	-	-
2024–7–16 (Day 6)	37.3	78	104/62	18	-	+	-	-
2024–7–17 (Day 7)	37.5	88	111/64	18	-	+	-	-
2024–7–18 (Day 8)	37.3	80	108/63	18	-	±	-	-
2024–7–19 (Day 9)	37.6	80	116/69	18	-	±	-	-
2024–7–20 (Day 10)	36.8	70	106/68	19	-	±	-	-
2024–7–21 (Day 11)	36.9	66	94/60	18	-	±	-	-
2024–7–22 (Day 12)	-	-	-	-	-	-	-	-
2024–7–23 (Day 13)	-	-	-	-	-	-	-	-
2024–7–24 (Day 14)	-	-	-	-	-	-	-	-
2024–7–25 (Day 15)	36.5	69	105/65	18	-	±	-	-
2024–7–26 (Day 16)	-	-	-	-	-	-	-	-
2024–7–27 (Day 17)	-	-	-	-	-	-	-	-
2024–7–28 (Day 18)	-	-	-	-	-	-	-	-

Temperature, Arterial pulse, Blood pressure, Respire values indicated are the highest measured for each day. The clinical symptoms of the patient in the early-stage include sudden high fever (39.0 – 41.0 ℃) with pain in the upper left abdomen. + present; - absent or normal. In the second half of the disease course, all clinical symptoms disappear.

Table S4. Changes in *Babesia microti* load under real-time fluorescence quantitative PCR

Gene	C_T1	C_T2	C_T3	Average C_T	ΔC_T	$\Delta\Delta C_T$	$2^{-\Delta\Delta CT}$
18S rRNA (July 12)	18.69	18.84	18.71	18.75	-0.47	0 [a]	1
GAPDH (July 12)	19.16	19.22	19.27	19.22			
18S rRNA (July 26)	35	35	35	35	17.74	18.21 [b]	0.00000329
GAPDH (July 26)	17.31	17.17	17.29	17.26			

GAPDH: Glyceraldehyde-3-phosphate dehydrogenase

ΔC_T= Average C_T of GAPDH - Average C_T of 18S rRNA

[a]$\Delta\Delta C_T$=ΔC_T of July 12 - ΔC_T of July 12,[b]$\Delta\Delta C_T$=ΔC_T of July 26 - ΔCT of July 12

$2^{-\Delta\Delta CT}$=Fold gene expression

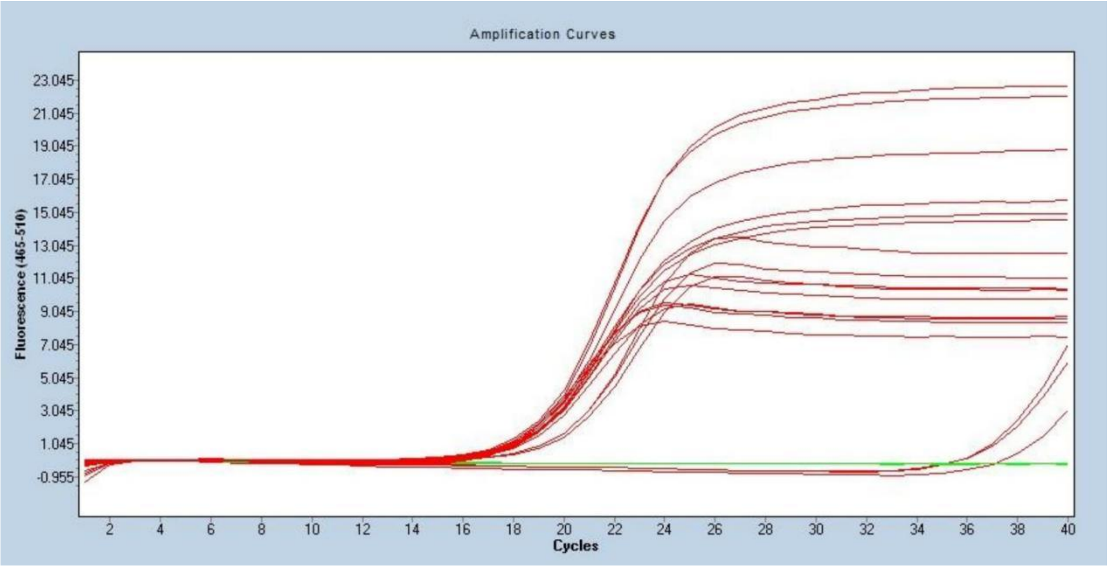

Roche LightCycler® 480 fluorescence quantitative pcr instrument threshold cycle number Ct value of the range of 15 − 35, if the measurement of the negative results, 35 for the measurement of the threshold, in order to carry out quantitative calculations, the negative results of the data is set as the limit of detection, so that the measured pathogens July 26 samples for the sample is July 12 samples for the 0.00000329 times, i.e., it is a reduction of $3.04x10^5$ times.
